# Influenza A Virus Superinfection Potential Is Regulated by Viral Genomic Heterogeneity

**DOI:** 10.1128/mBio.01761-18

**Published:** 2018-10-30

**Authors:** Jiayi Sun, Christopher B. Brooke

**Affiliations:** a Department of Microbiology, University of Illinois, Urbana, Illinois, USA; b Carl R. Woese Institute for Genomic Biology, University of Illinois, Urbana, Illinois, USA; Vanderbilt University Medical Center

**Keywords:** coinfection, influenza, superinfection, virus

## Abstract

Defining the specific factors that govern the evolution and transmission of influenza A virus (IAV) populations is of critical importance for designing more-effective prediction and control strategies. Superinfection, the sequential infection of a single cell by two or more virions, plays an important role in determining the replicative and evolutionary potential of IAV populations. The prevalence of superinfection during natural infection and the specific mechanisms that regulate it remain poorly understood. Here, we used a novel single virion infection approach to directly assess the effects of individual IAV genes on superinfection efficiency. Rather than implicating a specific viral gene, this approach revealed that superinfection susceptibility is determined by the total number of viral gene segments expressed within a cell. IAV particles that express a complete set of viral genes potently inhibit superinfection, while semi-infectious particles (SIPs) that express incomplete subsets of viral genes do not. As a result, virus populations that contain more SIPs undergo more-frequent superinfection. We further demonstrate that viral replicase activity is responsible for inhibiting subsequent infection. These findings identify both a major determinant of IAV superinfection potential and a prominent role for SIPs in promoting viral coinfection.

## Abstract (precis)

Superinfection, the sequential infection of a single cell by two or more virions, plays an important role in determining the replicative and evolutionary potential of influenza A virus (IAV) populations. The specific mechanisms that regulate superinfection during natural infection remain poorly understood. Here, we show that superinfection susceptibility is determined by the total number of viral genes expressed within a cell and is independent of their specific identity. Virions that express a complete set of viral genes potently inhibit superinfection, while the semi-infectious particles (SIPs) that make up the bulk of IAV populations and express incomplete subsets of viral genes do not. As a result, viral populations with more SIPs undergo more-frequent superinfection. These findings identify both the primary determinant of IAV superinfection potential and a prominent role for SIPs in promoting coinfection.

## INTRODUCTION

Influenza A viruses (IAV) are estimated to cause hundreds of thousands of deaths across the world every year during seasonal epidemics, despite widespread preexposure and vaccination (1). In addition to the yearly burden of seasonal influenza viruses, novel zoonotic IAV strains periodically emerge into humans from swine or birds, triggering unpredictable pandemics that can dramatically increase infection and mortality rates (2). Defining the specific factors that influence the evolution of influenza viruses is critical for designing more-effective vaccines, therapeutics, and surveillance strategies.

The prevalence of coinfection can play an enormous role in determining the replicative and evolutionary potential of IAV populations. This is a function of both the segmented nature of the viral genome and the enormous amount of genomic heterogeneity present within IAV populations (3, 4). Coinfection allows reassortment, i.e., the production of novel viral genotypes through the intermixing of the individual IAV genome segments (5, 6). Reassortment events have contributed to the emergence of every major influenza pandemic of the past century (7). Coinfection also facilitates the complementation and productive replication of the semi-infectious particles (SIPs) that make up the majority of IAV populations (8–12). Finally, increasing the frequency of coinfection can accelerate viral replication kinetics and virus output by increasing the average multiplicity of infection (MOI) (13–15). Thus, to better understand how IAV populations transmit and evolve, we must identify the specific host and viral factors that govern coinfection.

One of the primary means by which coinfection can occur is superinfection, the sequential infection of one cell by multiple viral particles. For some viruses, superinfection appears to occur freely (16, 17). In contrast, a diverse range of viruses actively inhibit superinfection through a variety of mechanisms, a phenomenon known as superinfection exclusion (SIE) (18–26). The only in-depth study of IAV superinfection performed to date concluded that the viral neuraminidase (NA) protein acts to potently and rapidly inhibit IAV superinfection by depleting infected cells of the sialic acid receptors required for viral entry (27). More recently, Dou et al. reported a narrow time window during which IAV superinfection was possible (13). These findings are consistent with recent studies that have argued that reassortment is rare during human infection (28, 29). However, the existence of a potent mechanism of IAV SIE is at odds with both the frequent coinfection observed in a variety of experimental settings and the widespread occurrence of reassortment at the global scale (30–35). Marshall et al. showed that superinfection occurring at up to 8 h after a primary infection leads to robust coinfection and reassortment in cell culture (36). Extensive coinfection and complementation have also been observed in the respiratory tracts of IAV-infected mice and guinea pigs (9, 37). Collectively, these results suggest that IAV superinfection can be restricted, but to what extent and through which specific mechanisms remain crucial open issues.

Here, we reveal that IAV superinfection potential is regulated by the extent of genomic heterogeneity within the viral population. We observed that superinfection susceptibility is influenced in a dose-dependent fashion by the number of viral genes expressed by the initially infecting virion. Further, we show that superinfection occurs more frequently in IAV populations with more SIPs than in those with fewer. Finally, we demonstrate that SIE is mediated by the presence of active viral replication complexes and is completely independent of the gene coding sequence. Taken together, our results reveal how genomic heterogeneity influences IAV superinfection potential and demonstrate how SIPs can modulate collective interactions within viral populations.

## RESULTS

### Influenza virus SIE occurs in multiple cell types and is independent of type I interferon (IFN) secretion.

A previous study of IAV SIE concluded that NA expression completely blocks susceptibility to superinfection by 6 h postinfection (hpi) (27). To explore the potential mechanisms of IAV SIE in greater detail, we developed a flow cytometry-based assay that allows us to precisely measure the effects of previous infection on superinfection efficiency. To clearly identify cells infected by the first virus or by the superinfecting virus or by both, we used two recombinant viruses that express antigenically distinct hemagglutinin (HA), NA, and NS1 proteins that we could distinguish using specific monoclonal antibodies (MAbs) that we had on hand (see Fig. S1 in the supplemental material). For the primary infection, we used a recombinant version of H1N1 strain A/Puerto Rico/8/34 (rPR8). For the secondary infection, we used a recombinant virus (rH3N2) that contained the HA and NA gene segments from H3N2 strain A/Udorn/72, the NS gene segment from A/California/04/09, and the remaining 5 segments from PR8.

10.1128/mBio.01761-18.1 FIG S1 Levels of expression of HA, NA, and NS1 by rPR8 and rH3N2 can be differentiated using specific MAbs. MDCK cells were infected with rPR8 or rH3N2 at an MOI of <0.3 TCID_50_/cell. At 19 hpi, cells were harvested, fixed, permeabilized, stained against H1 (H36-26), N1 (NA2-1C1), NS1 (1A7), and H3 (H14-A2), and run on an LSR II flow cytometer. The results of analyses of expression of H1 versus N1 and of NS1 versus H3 are shown in representative FACS plots. Download FIG S1, TIF file, 1.7 MB. Copyright © 2018 Sun and Brooke. 2018 Sun and Brooke. This content is distributed under the terms of the Creative Commons Attribution 4.0 International license.

**FIG 1:**
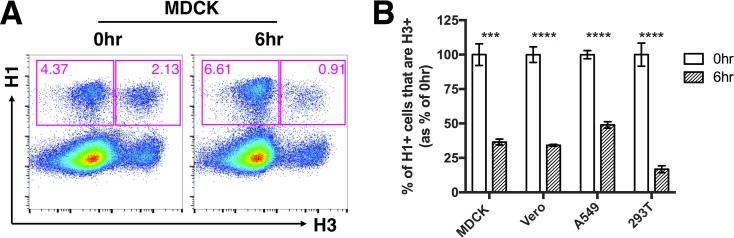
A 6-h delay between primary infection and superinfection allows robust superinfection exclusion. The indicated mammalian cell lines were infected with rPR8 virus and were simultaneously (0hr) or sequentially (6hr) infected with rH3N2 virus; all infections were performed at MOI = <0.3 TCID_50_/cell. (A) Representative fluorescence-activated cell sorter (FACS) plots showing expression of H1 versus H3 within MDCK cells. (B) H3^+^ frequencies within H1^+^ cells following simultaneous or sequential infection, in the indicated cell lines. The values for both the 0-h and 6-h groups are shown as percentages of the mean values of the 0-h control group to clearly illustrate the percentages of reduction in cellular susceptibility in the 6-h group. Data are presented as means (*n* = 3 cell culture wells) ± standard deviations. ***, P < 0.001; ****, P < 0.0001 (*t* test).

We first asked whether prior infection with rPR8 affected cellular susceptibility to superinfection with rH3N2. We infected Madin-Darby canine kidney (MDCK) cells with rPR8 at an MOI of <0.3 50% tissue culture infective doses (TCID_50_)/cell, and at 3 hpi (all times postinfection are expressed relative to the time at which the first virus was added) we added the PR8-HA-specific neutralizing MAb H17-L2 to block secondary spread of rPR8 within the culture. At 6 hpi, we infected with rH3N2 at an MOI of <0.3 TCID_50_/cell (6 hr). To prevent spread of both rPR8 and rH3N2, we added 20 mM NH_4_Cl at 9 hpi (38, 39). In parallel, we performed simultaneous coinfections (0 hr) with rPR8 and rH3N2 to measure coinfection frequencies under conditions SIE should not be possible. At 19 hpi, we harvested cells and examined primary and secondary virus infections by flow cytometry, using H1 expression and H3 expression as markers of rPR8 infection and rH3N2 infection, respectively. We observed that the H3-positive (H3^+^) frequency within H1^+^ cells was significantly reduced when rPR8 infection preceded rH3N2 by 6 h compared with when rPR8 and rH3N2 were added simultaneously (Fig. 1A). This indicated that rPR8 infection significantly reduces the susceptibility of cells to superinfection by 6 hpi.

We next asked whether the SIE effect was cell type specific and whether it depended on activation of the type I interferon (IFN) system. We performed the experiment described above in MDCK cells, A549 cells, human embryonic kidney HEK293T (293T), and Vero cells (which are incapable of type I IFN secretion) (40, 41). We observed that the levels of SIE were comparable among all cell lines tested, suggesting that SIE occurs in multiple distinct cell types and does not depend upon IFN secretion (Fig. 1B; see also Fig. S2).

10.1128/mBio.01761-18.2 FIG S2 Superinfection is inhibited in multiple cell lines. Levels of H1 expression versus H3 expression in Vero cells, A549 cells, and 293T cells observed in the experiments described in the Fig. 1 legend are shown as representative FACS plots. Download FIG S2, TIF file, 4.6 MB. Copyright © 2018 Sun and Brooke. 2018 Sun and Brooke. This content is distributed under the terms of the Creative Commons Attribution 4.0 International license.

### SIE does not depend upon viral neuraminidase activity.

**FIG 2:**
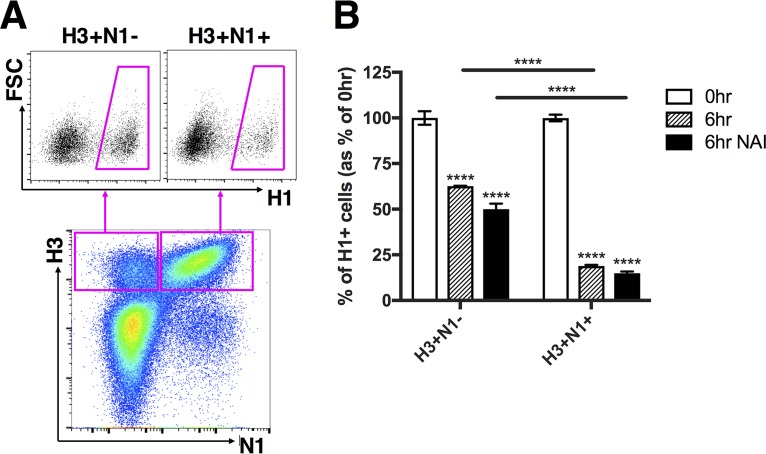
Superinfection exclusion is more potent in infected cells that express NA but is independent of NA enzymatic activity. MDCK cells were infected with rH3N1 virus and were simultaneously (0hr) or sequentially (6hr) infected with rH1N2 virus; all infections were performed at MOI = <0.3 TCID_50_/cell. During the 5-h gap and 1-h adsorption of the secondary infection (rH1N2), cells were incubated in either medium alone or media with 1 µM zanamivir (NAI). (A) Representative FACS plots comparing H1^+^ frequencies between H3^+^ N1^−^ and H3^+^ N1^+^ cells. (B) H1^+^ frequencies within H3^+^ N1^−^ and H3^+^ N1^+^ cells following simultaneous (0hr) or sequential (6hr) infection. Values of both the 0-h and 6-h groups (with or without the presence of NAI) are normalized to the means of 0-h controls, and data are presented as mean values (*n* = 3 cell culture wells) ± standard deviations. ****, P < 0.0001 (*t* test).

In an attempt to confirm the previously reported role for NA activity in SIE, we directly measured the effect of NA expression on SIE in our system (27). We took advantage of our previous observation that IAV populations consist primarily of SIPs that fail to express one or more viral genes (8). When carrying out the primary infection at a low MOI, we generate populations of infected cells that are either positive or negative for expression of a given viral gene. We can then assess the effects of specific viral proteins on superinfection susceptibility by comparing superinfection frequencies between infected cells that do or do not express the protein in question (Fig. 2A).

We performed the same superinfection experiment as described above in MDCK cells; however, we used slightly different viruses to ensure that the NA specificity of the primary virus was well matched to the HA specificity of the secondary virus. The primary virus used here encoded the HA gene from A/Udorn/72 and the NA gene from PR8 (rH3N1), while the secondary virus encoded the HA gene from PR8 and the NA gene from A/Udorn/72 (rH1N2). The remaining 6 segments for both viruses came from PR8.

At 19 hpi, we harvested and stained with MAbs against H1, N1, and H3. To compare rH3N1 infected cells that did or did not express NA, we individually gated cells into H3^+^ N1^+^ and H3^+^ N1^−^ subpopulations (Fig. 2A). Comparison of H1^+^ frequencies between H3^+^ N1^+^ and H3^+^ N1^−^ cells revealed that NA expression in infected cells was clearly associated with decreased susceptibility to superinfection (Fig. 2B). This finding was consistent with the previously reported role for NA in IAV superinfection exclusion (27). Importantly, while SIE was more pronounced in the H3^+^ N1^+^ cells, we also observed a significant decrease in superinfection susceptibility within the H3^+^ N1^−^ cell population by 6 hpi, suggesting that viral factors other than NA also act to restrict superinfection.

To directly test whether NA enzymatic activity was required for the increased potency of SIE that we observed in NA^+^ cells, we asked whether treatment with the NA inhibitor (NAI) zanamivir could diminish this effect. We observed that the addition of zanamivir at 1 μM (a concentration that completely blocked rH3N1 NA enzymatic activity) did not decrease the strength of SIE in NA^+^ cells (Fig. 2B; see also Fig. S3). We also examined the effects of two substitutions (NP:F346S and NA:K239R) that decrease cellular NA expression relative to wild-type PR8 on superinfection efficiency (9, 42) (Fig. S4A). In accordance with our zanamivir results, these mutants did not exhibit higher superinfection frequencies than wild-type PR8 (Fig. S4B). Taken together, these results reveal that superinfection is more effectively inhibited by virions that express NA than by those that do not but that NA enzymatic activity is dispensable for this effect.

10.1128/mBio.01761-18.3 FIG S3 Viral NA activity is blocked by 1 µM zanamivir. (A) NA activity of rH3N1 virus used in the experiment described in the Fig 2 legend. Data represent results of determinations of relative fluorescence units (RFU) against time under conditions of increasing concentrations of zanamivir. (B) *V*_max_ of reactions performed as described for panel A. Data are presented as mean values (*n* = 2 enzymatic reactions) ± standard deviations. Download FIG S3, TIF file, 0.8 MB. Copyright © 2018 Sun and Brooke. 2018 Sun and Brooke. This content is distributed under the terms of the Creative Commons Attribution 4.0 International license.

10.1128/mBio.01761-18.4 FIG S4 Superinfection exclusion is independent of NA expression level. MDCK cells were infected with rPR8_WT_, rPR8_NP:F346S_, or rPR8_NA:K239R_ and were simultaneously (0hr) or sequentially (6hr) infected with rH3N2; all infections were performed at MOI = <0.3 TCID_50_/cell. (A) Comparison of NA expression levels within H1^+^ N1^+^ cells between rPR8_WT_, rPR8_NP:F346S_, and PR8_NA:K239R_ at 19 hpi_._ (B) Comparison of H3^+^ frequencies within H1^+^ N1^−^ and H1^+^ N1^+^ cells between the three indicated viruses. Values from h 6 groups are normalized to means of h 0 controls. Data are presented as mean values (*n* = 3 cell culture wells) ± standard deviations. Download FIG S4, TIF file, 1.5 MB. Copyright © 2018 Sun and Brooke. 2018 Sun and Brooke. This content is distributed under the terms of the Creative Commons Attribution 4.0 International license.

### Superinfection susceptibility is determined by the number of viral genes expressed in the cell.

**FIG 3:**
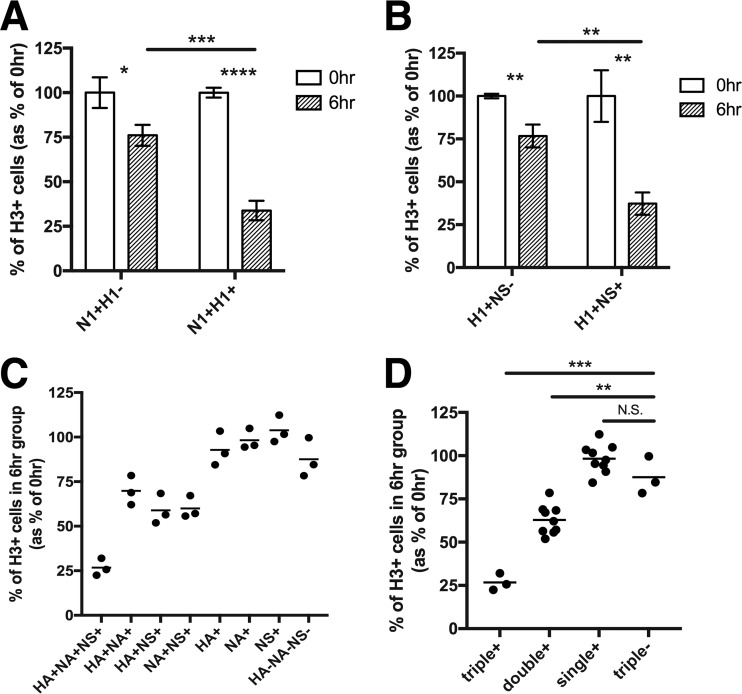
Superinfection is more frequent in cells that express fewer viral genes. MDCK cells were infected with rPR8 and were simultaneously (0hr) or sequentially (6hr) infected with rH3N2; all infections were performed at MOI = <0.3 TCID_50_/cell. (A and B) Representative FACS plots comparing H3^+^ frequencies between (A) N1^+^ H1^−^ and N1^+^ H1^+^ cells and (B) H1^+^ NS^−^ and H1^+^ NS^+^ cells. (C) Superinfection assay performed as described for panels A and B; however, cells are gated into all 8 possible combinations of HA, NA, and NS1 expression by rPR8, and H3^+^ percentages are compared between these subpopulations. Data represent the values of 6hr groups normalized to the means of 0hr controls. Each data point represents the H3^+^ frequency for the indicated cell population within a single cell culture well, shown as a percentage of the mean H3^+^ frequency of the same cell subpopulation in the 0hr controls. (D) Data from panel C grouped by total numbers of the three examined viral gene products (HA, NA, and NS1) expressed by rPR8 rather than by their specific identities. *, P < 0.05; **, P < 0.01; ***, P < 0.001; ****, P < 0.0001; N.S., not significant (*t* test).

On the basis of our observation that superinfection was also inhibited within N1^−^ cells (Fig. 2B), we hypothesized that expression of other viral gene products might also inhibit superinfection. We examined the effects of HA and NS1 expression on superinfection susceptibility, using rPR8-specific MAbs. Surprisingly, we found that both HA expression and NS1 expression within rPR8-infected cells were associated with significant decreases in superinfection by rH3N2 and that the results were comparable to the effect associated with NA expression (Fig. 3A and B). To further dissect how viral gene expression patterns influence SIE, we individually gated all seven possible combinations of HA, NA, and NS1 expression by rPR8 (HA^+^ NA^+^ NS1^+^, HA^+^ NA^+^, HA^+^ NS1^+^, NA^+^ NS1^+^, HA^+^, NA^+^, NS1^+^) and directly compared their rH3N2 infection frequencies (the gating scheme is shown in Fig. S5A). We observed that the fraction of cells superinfected with rH3N2 was inversely correlated with the number of rPR8 genes expressed (among the three we examined), regardless of their specific identities (Fig. 3C and D). Thus, susceptibility to IAV superinfection is determined by the number of viral genes expressed in the host cell by the initially infecting virion.

10.1128/mBio.01761-18.5 FIG S5 Gating scheme used for measuring the effect of the number of viral genes expressed on superinfection frequencies. (A) Cells from the experiment described in the Fig. 3 legend were assessed for expression of HA, NA, and NS1 sequentially. The fractions of H3^+^ cells (indicative of superinfection rates) were quantified and compared between the cell populations with the indicated viral gene expression patterns. (B) Representative FACS plots showing the gates used to assess HA, NA, and NS1 expression in this experiment. Download FIG S5, TIF file, 3.1 MB. Copyright © 2018 Sun and Brooke. 2018 Sun and Brooke. This content is distributed under the terms of the Creative Commons Attribution 4.0 International license.

### Superinfection is more prevalent in IAV populations with more SIPs.

If the number of viral genes expressed in a cell determines superinfection susceptibility, then decreasing the average number of functional viral genes successfully delivered by individual virions should increase the overall incidence of superinfection. We tested this by artificially decreasing the functional gene segment content of rPR8 through exposure to UV irradiation (43). Exposure to low-dose UV irradiation generates SIPs that carry gene-lethal UV-induced lesions at frequencies proportional to genome segment length. On the basis of our previous findings, we hypothesized that superinfection frequencies would increase with longer exposure of rPR8 to UV.

**FIG 4:**
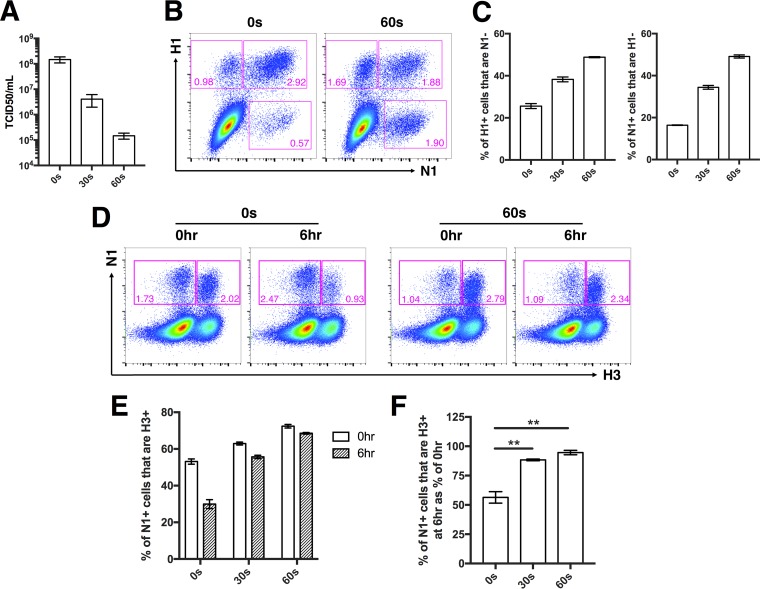
Superinfection is more common in viral populations that contain more SIPs. rPR8 was irradiated by the use of a 302-nm-wavelength UV lamp for 30 s or 60 s. (A) TCID_50_ titers of untreated rPR8 (0 s) or rPR8 subjected to UV treatment for 30 s and 60 s. Pooled data from two independent experiments are shown. (B) Representative FACS plots showing HA versus NA expression patterns of untreated rPR8 (0 s) and rPR8 subjected to UV treatment for 60 s. (C) Quantification of H1^−^ and N1^−^ SIPs in untreated rPR8 (0 s) or rPR8 subjected to UV treatment for 30 s and 60 s. (D) MDCK cells were infected with untreated rPR8 (0 s) or with rPR8 subjected to UV treatment for 30 s and 60 s at an MOI of 0.04 NAEU/cell and were simultaneously (0hr) or sequentially (6hr) infected with rH3N2 at MOI < 0.3 TCID_50_/cell. Representative FACS plots showing expression of N1 versus H3 in cells infected with untreated rPR8 (0 s) or with rPR8 subjected to UV treatment for 60 s. (E) rH3N2 infection percentages within N1^+^ cells infected with untreated rPR8 (0 s) or with rPR8 subjected to UV treatment for 30 s and 60 s. (F) Values corresponding to the 6hr groups in panel E normalized to means of 0hr controls. For panels E and F, data are presented as mean values (*n* = 3 cell culture wells) ± standard deviations. **, P < 0.01 (*t* test).

We used UV (302-nm wavelength) to irradiate rPR8 for either 30 s or 60 s and confirmed that the TCID_50_ concentration was reduced and the SIP concentration was increased as a function of treatment duration (Fig. 4A to C). We then performed superinfection assays as described above, comparing rH3N2 superinfection frequencies following infection by untreated or UV-irradiated rPR8 in MDCK cells. To fairly compare superinfection frequencies between viral populations with differing particle-to-infectivity ratios, we normalized our rPR8 infections based on equivalent numbers of particles capable of expressing NA (NA-expressing units [NAEU]) (9).

We first examined the effect of UV treatment on superinfection when rPR8 and rH3N2 were added to cells simultaneously (at h 0). This was a critical control because UV treatment can increase the measured incidence of coinfection, independently of SIE effects, purely by creating a larger pool of SIPs that show up in our assays only when complemented by secondary infection (43). Consistent with this, we observed a small increase in coinfection frequency with UV treatment when the two viruses were added simultaneously (Fig. 4D and E). When rH3N2 was added 6 h after rPR8, however, we observed a much more pronounced increase in superinfection frequency with UV treatment, consistent with our hypothesis that superinfection can be regulated by the proportion of SIPs present within the viral population (Fig. 4D to F).

### SIE is mediated by active IAV replication complexes and is independent of the gene coding sequence.

Our data reveal that IAV superinfection potential is determined by the number of viral genes expressed within a cell, independent of their specific identity. This suggests that the viral gene products themselves are dispensable for SIE. We thus hypothesized that active replication and/or transcription of viral RNAs by the viral replicase complex might be responsible for decreasing cellular susceptibility to subsequent infection. To test this, we cotransfected 293T cells with pDZ vectors encoding the individual viral replicase proteins (PB2, PB1, PA, and NP) together with a pHH21 vector encoding either the HA vRNA gene segment (HA_vRNA_) or a vRNA-derived reporter gene segment in which the enhanced green fluorescent protein (eGFP) open reading frame (ORF) is flanked by the 5′ and 3′ untranslated-region (UTR) sequences from the NA segment (eGFP_vRNA_). These UTR sequences are required for replication and transcription of the reporter RNA by the viral replicase. At 24 h posttransfection, we infected cells with rH3N2 at an MOI of 0.2 TCID_50_/cell and measured infectivity at 8 hpi using an M2-specific MAb.

**FIG 5:**
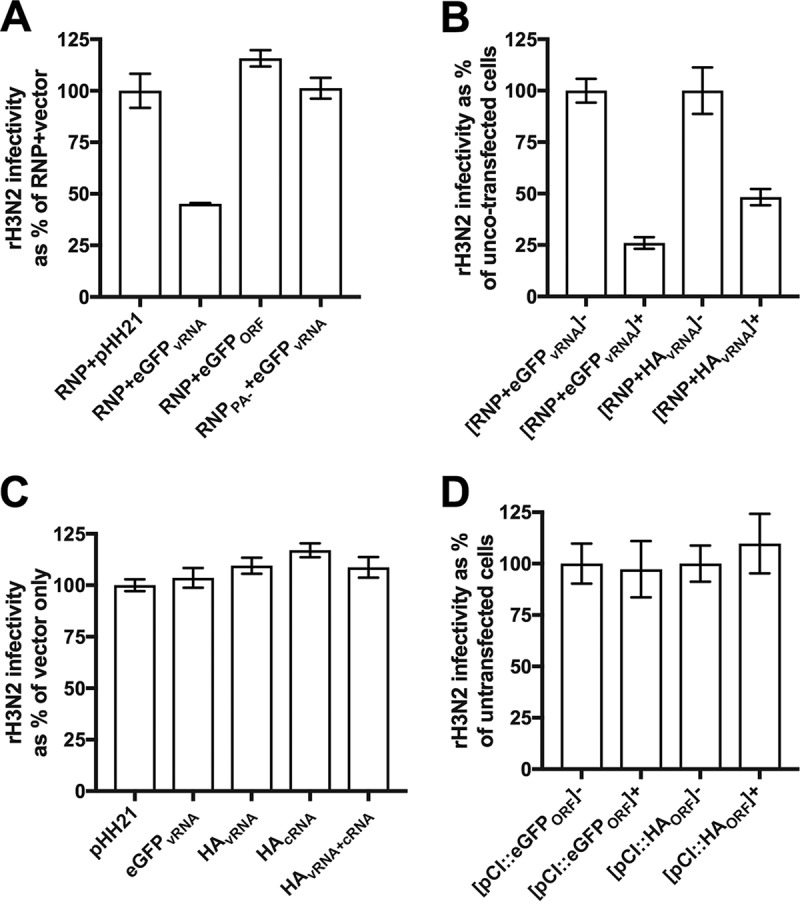
Viral replicase activity inhibits subsequent infection. (A) Subconfluent 293T cell monolayers were transfected with: plasmids encoding the complete set of viral replicase proteins (PB2, PB1, PA, and NP) along with a plasmid that encodes a reporter vRNA (eGFP ORF plus 5′ and 3′ viral UTR sequences; pHH21::eGFP_vRNA_) under the control of the polymerase I (PolI) promoter (RNP + eGFP_vRNA_), plasmids encoding the complete set of viral replicase proteins along with a plasmid (pHH21::eGFP_ORF_) that encodes the eGFP ORF without the viral UTRs (RNP + eGFP_ORF_), plasmids encoding an incomplete set of three viral replicase proteins (PB2, PB1, and NP only; no PA) along with the viral reporter RNA plasmid pHH21::eGFP_vRNA_ (RNP_PA-_ + eGFP_vRNA_), or plasmids encoding the complete set of viral replicase proteins along with an empty vector control (RNP + pHH21). At 24 h posttransfection, cells were infected with rH3N2 at MOI = 0.2 TCID_50_/cell. At 8 hpi, cells were harvested and assessed for rH3N2 infection via flow cytometry. For each group, rH3N2 infectivities are presented as the percentages of the mean rH3N2 infectivity in the RNP + pHH21 vector control group. (B) Experiment performed as described for panel A with cotransfection of plasmids encoding the complete set of viral replicase proteins plus either pHH21::eGFP_vRNA_ or pHH21::HA_vRNA_. Data representing rH3N2 infectivity in cotransfected cells (eGFP^+^ or HA^+^) are normalized to uncotransfected cells (eGFP^−^ or HA^−^) in the same samples. (C) Experiment performed as described for panel A with transfection of pHH21 vector, pHH21::eGFP_vRNA_, pHH21::HA_vRNA_, pHH21::HA_cRNA_, or pHH21::HA_vRNA_ together with pHH21::HA_cRNA_. rH3N2 infectivities are presented as the percentages of the mean rH3N2 infectivity in the vector control group. (D) Experiment performed as described for panel A with transfection of pCI vector, pCI::eGFP_ORF_, or pCI::HA_ORF_. Data representing rH3N2 infectivity in transfected cells (eGFP^+^ or HA^+^) are normalized to untransfected cells (eGFP^−^ or HA^−^) in the same samples. Data are presented as mean values (*n* = 2 cell culture wells) ± standard deviations.

Infection frequencies were decreased ∼50% in cells expressing the replicase components plus the eGFP_vRNA_ construct compared with control cells transfected with the replicase-expressing constructs plus an empty pHH21 vector (Fig. 5A). In analyses of differences in the levels of rH3N2 infectivity between cotransfected cells (eGFP^+^, HA^+^) and uncotransfected cells (eGFP^−^, HA^−^) within the same culture wells, the inhibitory effects mediated by eGFP_vRNA_ or HA_vRNA_ expression were found to be comparable (Fig. 5B; see also Fig. S6). Importantly, this effect was not seen when we left out the plasmid encoding PA (RNP_PA−_) or used an eGFP reporter RNA that lacked the viral UTR sequences (eGFP_ORF_) (Fig. 5A). Taken together, these data indicate that inhibition of infection requires both an intact replicase complex and an RNA template containing the viral UTR sequences but not the viral coding sequence.

10.1128/mBio.01761-18.6 FIG S6 Cells cotransfected with plasmids encoding viral replicase and viral RNA are less susceptible to subsequent infection. Cells from the experiments described in the Fig. 5A and B legends were assessed for expression of M2 (indicative of rH3N2 infection) versus eGFP and HA (indicative of cotransfection) as shown in representative FACS plots. Download FIG S6, TIF file, 3.6 MB. Copyright © 2018 Sun and Brooke. 2018 Sun and Brooke. This content is distributed under the terms of the Creative Commons Attribution 4.0 International license.

Our data demonstrate that IAV SIE is mediated by the specific activity of viral replication complexes. One potential explanation is that large amounts of recently synthesized negative-sense vRNA within the cell might outcompete incoming genome segments for replication and expression. To test this, we transfected 293T cells with a pHH21 vector that overexpresses the eGFP_vRNA_ segment and measured susceptibility to rH3N2 infection 24 h later using an NP-specific MAb. Compared to the empty vector control, we observed no effect of eGFP_vRNA_ vRNA overexpression on cellular susceptibility to infection (Fig. 5C). Similarly, we observed no effect when we overexpressed the cRNA and vRNA forms of the HA gene segment, either individually or together (Fig. 5C). It must be noted that bulk levels of viral RNA were roughly 5-fold lower in these cells than in cells that express the viral replicase, so we cannot rule out a role for the intracellular abundance of viral RNA as a determinant of susceptibility to subsequent infection (Fig. S7).

10.1128/mBio.01761-18.7 FIG S7 Comparison of intracellular viral RNA levels measured under different experimental conditions. (A) Relative amounts of HA vRNA in 293T cells transfected with pHH21::HA_vRNA_ or cotransfected with plasmid-derived viral replicase complex plus pHH21::HA_vRNA_ for 24 h or infected with rPR8 for 6 h at MOI = 0.5 HAEU/cell were determined by strand-specific quantitative real-time PCR, including the no-reverse-transcription control. Data shown are delta threshold cycle (Δ*C_*T*_*) values for HA vRNA versus GAPDH mRNA. (B) Relative amounts of HA viral RNA shown as fold differences compared with HA_vRNA_. Data are presented as mean values (*n* = 2 cell culture wells) ± standard deviations. Download FIG S7, TIF file, 1.1 MB. Copyright © 2018 Sun and Brooke. 2018 Sun and Brooke. This content is distributed under the terms of the Creative Commons Attribution 4.0 International license.

Another potential explanation is that viral mRNA or protein overexpression might inhibit subsequent infection. To test this, we transfected 293T cells with pCI vectors that overexpress mRNA and protein of eGFP and HA and measured susceptibility to rH3N2 infection 24 h posttransfection using the M2-specific MAb. Compared to the empty vector control, mRNA/protein overexpression of eGFP or HA had no effect on infection susceptibility (Fig. 5D; see also Fig. S8). Collectively, these data demonstrate that IAV superinfection exclusion is triggered by the activity of viral replication complexes.

10.1128/mBio.01761-18.8 FIG S8 Overexpression of viral mRNA and protein in cells does not inhibit subsequent infection. (A) Cells from the experiment described in the Fig. 5D legend were assessed for expression of M2 (indicative of rH3N2 infection) versus eGFP and HA (indicative of transfection) in representative FACS plots. (B) rH3N2 infectivity data from panel A are shown as percentages of pCI vector. Data are presented as mean values (*n* = 2 cell culture wells) ± standard deviations. Download FIG S8, TIF file, 2.9 MB. Copyright © 2018 Sun and Brooke. 2018 Sun and Brooke. This content is distributed under the terms of the Creative Commons Attribution 4.0 International license.

## DISCUSSION

Superinfection plays an enormous role in influencing the outcome of IAV infection, both by promoting reassortment and by facilitating the multiplicity reactivation of SIPs and defective interferring particles (4). Despite this importance, the specific factors that govern the occurrence of superinfection have remained obscure. Here, we reveal that IAV superinfection susceptibility is regulated by the number of viral genes successfully expressed by a virion. We further demonstrate that the presence of SIPs within viral populations significantly increases the frequency of superinfection. This represents a completely novel mechanism of viral superinfection exclusion and identifies a clear functional consequence of the enormous genomic heterogeneity within IAV populations.

The only other published study that examined IAV SIE in detail concluded that NA expression mediates SIE by depleting the pool of available sialic acid receptors on the cell surface (27). In this study, we directly quantified the contribution of NA expression to SIE during IAV infection and found that the SIE effect of NA expression is actually comparable to that of other viral genes. We also show that treatment with NAIs has no appreciable effect on superinfection susceptibility. The conclusions of the study by Huang et al. were based primarily on two observations: (i) overexpression of NA within cells rendered them refractory to infection by an HA-pseudotyped virus, and (ii) IAV superinfection occured only when cells were treated with NAIs (27). While we cannot conclusively explain the discrepancies between the two studies, we can offer a couple of plausible explanations. First, the cellular overexpression studies in Huang et al. likely involved levels of cellular NA expression that are far beyond those seen during IAV infection. Second, the observation that NAI treatment dramatically increases superinfection frequencies may be explained by the effects of cell death. In their experiments, Huang et al. infected cells at a relatively high MOI, did not block secondary spread of the virus within cultures, and assessed superinfection frequency at 20 hpi or later. Under those conditions, many of the initially infected cells may have been dead or dying and thus lost from the analysis. That may be especially true of superinfected cells, which tend to be infected at a higher than the average effective MOI. Even under low-MOI conditions, we had to limit the time frame of our experiments and block secondary spread of virus to prevent cell death from skewing our results. NAI treatment may act to help preserve coinfected cells so that they are detected at the endpoint of the experiment, thus increasing the measured superinfection rate.

Our results reveal that SIE is triggered in a dose-dependent fashion by the number of functional viral gene segments delivered by the initially infecting virion. The surprising irrelevance of the specific IAV gene segments involved is explained by our finding that the viral coding sequence of a gene segment can be replaced with that of eGFP without any loss of inhibitory effect. This suggests a direct role for viral replicase activity itself in triggering SIE rather than any effect of the viral gene segments themselves. We hypothesize that the overall levels of replicase activity in the cell, particularly during the early stages of infection, are determined by the number of functional RNPs delivered to the cell. Thus, cells infected by SIPs that deliver fewer functional RNPs exhibit lower overall levels of replicase activity, resulting in less-potent SIE.

The specific mechanism by which the activity of viral replicase complexes may inhibit subsequent infection remains unclear; however, one potential explanation is that the activity of viral replication complexes triggers a cell-intrinisic antiviral response. As each incoming gene segment brings its own replicase complex, this could potentially explain the gene dose-dependent suppression of superinfection that we observed. While our experiments in Vero cells demonstrated that the secretion of type I IFN is not required for SIE, they do not preclude the involvement of type I IFN-independent mechanisms. These could include either the type III IFN-mediated induction of antiviral effectors or the engagement of completely IFN-independent antiviral mechanisms (44–46). Future studies will be aimed at delineating the role of the host in the regulation of IAV superinfection.

Our results demonstrate that SIPs can directly influence the prevalence of superinfection and thus, potentially, the frequency of reassortment. Fonville et al. used a UV irradiation-based method similar to that shown here to demonstrate that increasing the frequency of SIPs within a viral popuation increases the overall reassortment rate (43). The explanation given for this effect was that increasing the abundance of SIPs increases the proportion of the viral population that depends upon coinfection to replicate. As a result, within a certain MOI range, a greater share of productively infected cells are coinfected and subject to reassortment. In our study, we confirmed this effect by observing a slight increase in coinfection frequency with increasing UV dose when rPR8 and rH3N2 were added simultaneously (Fig. 4E). When we controlled for this, however, we still observed a significant increase in superinfection frequency as we increased the proportion of SIPs through UV treatment (Fig. 4D to F). Thus, the relationship between SIPs and SIE that we describe here is completely independent of the increased multiplicity reactivation observed by Fonville et al. and likely represents the effects of decreasing the strength of SIE. Between these two studies, it is clear that SIPs can modulate the frequency of IAV coinfection and reassortment through two distinct mechanisms.

IAV strains can differ significantly with respect to the relative production and gene expression patterns of SIPs (8, 9). This raises the possibility that strains with distinct SIP production phenotypes may differ in their reassortment potential, given the influence of SIPs on coinfection and reassortment frequencies. If this is the case, it would suggest a significant role for production of SIPs in governing the evolutionary potential of IAV populations.

The relationship between viral gene expression patterns and superinfection exclusion that we report here demonstrates that viral genomic heterogenity has distinct functional consequences during infection. A crucial implication is that all infected cells cannot be thought of as equal but may in fact exhibit distinct phenotypes based on the number and identity of viral genome segments that they harbor (47). The relationship between viral genomic heterogeneity and the phenotypic diversity of infected cells likely extends to cellular features beyond superinfection susceptibility.

It remains to be seen whether the relationship between viral gene dose and superinfection susceptibility that we describe here exists for other segmented viruses besides IAV. Beyond the segmented viruses, it has become increasingly clear that collective interactions mediated by cellular coinfection significantly influence the replicative and evolutionary dynamics of nonsegmented viruses as well (48). More work is needed to better understand the factors that govern coinfection for different virus families, both *in vitro* and *in vivo*.

In summary, our work reveals a unique mechanism of IAV superinfection regulation that is governed by viral genomic heterogeneity. Critically, we show that the abundance of SIPs within a viral population directly influences the prevalence of superinfection; suggesting that IAV strains may differ in their superinfection potential and thus in their potential for reassortment. This finding has significant consequences for understanding how SIP production can influence the evolutionary potential of IAV populations. More broadly, we demonstrate how genomic diversity within viral populations can have clear functional consequences during infection.

## MATERIALS AND METHODS

### Plasmids.

The A/Puerto Rico/8/34 and A/Udorn/72 reverse genetics plasmids were generous gifts from Adolfo Garcia-Sastre and Kanta Subbarao, respectively. The pCI vector was graciously provided by Joanna Shisler. pHH21::eGFP_vRNA_ (eGFP ORF flanked by the NA UTRs) was kindly gifted by Andrew Mehle. Generation of pHH21::HA_vRNA_ has been previously described (9). The following primer pairs were used to generate the indicated constructs: for pHH21::eGFP_ORF_ (BsmBI), 5′-CGTCTCCTATTTTACTTGTACAGCTCG and 3′ CGTCTCCGGGATGGTGAGCAAGGGC; for pHH21::HA_cRNA_ (BsmBI), 5’CGTCTCATATTAGCAAAAGCAGG and 3′ CGTCTCAGGGAGTAGAAACAAGGG; for pCI::eGFP_ORF_ (EcoRI/SalI), 5′-AGAATTCATGGTGAGCAAGG and 3’AGTCGACTTACTTGTACAGC; for pCI::HA_ORF_ (EcoRI/SalI), 5′-AGAATTCATGGAAGATTTTGTGCG and 3′ AGTCGACCTAACTCAATGCATGTGT.

### Cells.

Madin-Darby canine kidney (MDCK) and human embryonic kidney HEK293T (293T) cells were maintained in Gibco’s minimal essential medium (MEM) with GlutaMax (Life Technologies). Vero cells were maintained in Dulbecco’s modified Eagle medium (Life Technologies). Human lung epithelial A549 cells were maintained in Gibco’s F-12 medium (Life Technologies). MDCK, Vero, and A549 cells were obtained from Jonathan Yewdell; 293T cells were obtained from Joanna Shisler. All media were supplemented with 8.3% fetal bovine serum (Seradigm). Cells were grown at 37°C and 5% CO_2_.

### Viruses.

Recombinant A/Puerto Rico/8/1934 (rPR8) and rH3N2 viruses were generated using 8-plasmid rescue systems. The rH3N2 virus is a reassortant with the HA and NA segments from A/Udorn/72 (H3N2), the NS segment from A/California/04/09 (H1N1), and the other 5 segments from PR8. The rPR8 clones differ from the published sequence (GenBank accession no. AF389115 to AF389122) at two positions: PB1 A549C (K175N) and HA A651C (I207L) (numbering from initiating Met). Molecular clone-derived mutants (rPR8 NP:F346S and rPR8 NA:K239R) were generated using standard site-directed PCR mutagenesis. All viruses were rescued by transfecting subconfluent 293T cells with 500 ng of each of the appropriate reverse genetics plasmids using JetPRIME (Polyplus) according to the manufacturer’s instructions. Plaque isolates derived from rescue supernatants were amplified into seed stocks in MDCK cells. Working stocks were generated by infecting MDCK cells at an MOI of 0.0001 TCID_50_/cell with seed stock and collecting and clarifying supernatants at 48 hpi. All viral growth was carried out in MEM with 1 μg/ml trypsin treated with l-(tosylamido-2-phenyl) ethyl chloromethyl ketone (TPCK-treated trypsin; Worthington), 1 mM HEPES, and 100 μg/ml gentamicin. The titers of the virus stocks were determined via standard 50% tissue culture infectious dose (TCID_50_) assay.

### Superinfection assay.

For the 6-h sequential-infection group, confluent mammalian cells (MDCK, Vero, A549, or 293T) in six-well plates were infected with rPR8 at an MOI of <0.3 TCID_50_/cell for 1 h. At 1 h postadsorption, monolayers were washed with phosphate-buffered saline (PBS) and incubated in serum-containing medium. At 3 hpi, neutralizing anti-PR8-HA mouse MAb H17-L2 (5 μg/ml) was added to cultures to prevent spread of rPR8. At 6 hpi, monolayers were superinfected with rH3N2 at an MOI of <0.3 TCID_50_/cell in the presence of H17-L2 (which does not interfere with rH3N2 infection) (see Fig. S2 in the supplemental material). At 1 h postadsorption, monolayers were washed with PBS and incubated in serum-containing medium with H17-L2. At 9 hpi of rPR8 (3 hpi of rH3N2), the medium was changed to MEM with 50 mM HEPES and 20 mM NH_4_Cl to block spread of both viruses. At 19 hpi of rPR8 (13 hpi of rH3N2), monolayers were trypsinized into single-cell suspensions.

For the 0-h simultaneous infection group, cells were infected with a mixture of rPR8 and rH3N2 at the same MOIs as in 6-h superinfection group. The NH_4_Cl medium was added to block viral spread at 3 hpi, and cells were harvested at 19 hpi.

All cells were simultaneously fixed and permeabilized using foxP3 fix/perm buffer (eBioscience). Fixed cells were stained with Alexa Fluor 488-conjugated mouse anti-H1 MAb H36-26 (which does not compete with H17-L2), Pacific Orange-conjugated mouse anti-N1 MAb NA2-1C1, Pacific Blue-conjugated mouse anti-NS1 MAb NS1-1A7, and Alexa Fluor 647-conjugated mouse anti-H3 MAb H14-A2 (all MAbs were gifts from Jon Yewdell). After staining was performed, cells were washed, run on a BD LSR II flow cytometer, and analyzed using FlowJo version 10.1 (Tree Star, Inc.). Importantly, the expression patterns of H1, H3, N1, and NS1 allowed clear gating of positive and negative populations.

### NA activity inhibition assay.

The NA inhibitor zanamivir (Sigma) (10 μg/ml) was 2-fold serially diluted in assay buffer [33 mM 2-(N-morpholino)ethane-sulfonic acid (pH 6.5), 4 mM CaCl_2_] and mixed with 2.2 × 10^6^ TCID_50_ of rH3N1 virus in a final volume of 50 μl. On a 96-well half-well flat-bottom plate (Corning 3686), 25 μl of the virus-NAI mixture was mixed with 20 μl of the fluorogenic substrate MUNANA [2′-(4-methylumbelliferyl)-α-d-N-acetylneuraminic acid] (Sigma) diluted in assay buffer [33 mM 2-(N-morpholino)ethane-sulfonic acid (pH 6.5), 4 mM CaCl_2_]. In the mixture, the final concentration was 200 μM for the substrate and ranged from 0.0625 μM to 1 μM for zanamivir. No-drug controls and no-virus controls were also included. Immediately after the addition of the substrate, fluorescence was measured every 5 min over a 30-min period on a SpectraMax M2 microplate reader (Molecular Devices). Michaelis-Menten kinetics were determined for each dilution of zanamivir to estimate the maximum rate of metabolism (*V*_max_) values.

### Quantification of NA expression.

H1^+^ N1^+^ MDCK cells infected with rPR8WT, rPR8 NP:F346S, or rPR8 NA:K239R in the superinfection assay (6-h group) were gated, and histograms for NA expression were plotted. The geometric mean fluorescence intensities (GMFI) for NA were determined using FlowJo version 10.1 (Tree Star, Inc.).

### UV treatment and analysis.

rPR8 stocks were placed in six-well plates (500 μg/well) on ice. Plates were placed 5 cm underneath a 302-nm-wavelength UVP-57 handheld UV lamp (UVP) and irradiated for 30 s or 60 s. TCID_50_ titers and single virion expression patterns of untreated and UV-treated virus were determined on MDCK cells, and the superinfection assays described above were performed using these viruses and rH3N2.

### Transfection assay.

293T cells (80% confluent) in six-well plates were transfected with the following plasmids using jetPRIME (Polyplus): RNP (500 ng each of pDZ::PB2, pDZ::PB1, pDZ::PA, and pDZ::NP) plus 1 μg of pHH21 vector; RNP plus 1 μg of pHH21::eGFP_vRNA_ (eGFP_ORF_ flanked with NA UTRs); RNP plus 1 μg of pHH21::eGFP_ORF_; RNP plus 1 μg of pHH21::HA_vRNA_; RNP_PA−_ (500 ng each of pDZ::PB2, pDZ::PB1, pDZ, and pDZ::NP) plus 1 μg of pHH21::eGFP_vRNA_; RNP_PA−_ plus 1 μg of pHH21::HA_vRNA_; 6 μg of pHH21 vector; 3 μg of pHH21 vector plus 3 μg of pHH21::eGFP_vRNA_; 3 μg of pHH21 vector plus 3 μg of pHH21::HA_vRNA_; 3 μg of pHH21 vector plus 3 μg of pHH21::HA_cRNA_; 3 μg of pHH21::HA_vRNA_ plus 3 μg of pHH21::HA_cRNA_; 3 μg of the pCI vector; 3 μg of pCI::eGFP_ORF_; 3 μg of pCI::HA_ORF_. All plasmid-encoded viral sequences were derived from PR8. At 24 h posttransfection, monolayers were infected with rH3N2 at an MOI of 0.2 TCID_50_/cell. At 8 hpi, cells transfected by RNP^+^ pHH21, and pCI plasmids were harvested and stained with Alexa Fluor 488-conjugated mouse anti-H1 MAb H36-26 and Alexa Fluor 647-conjugated mouse anti-M2 MAb O19. Cells transfected with pHH21 plasmids were permeabilized, fixed, and stained with Alexa Fluor 647-conjugated mouse anti-NP MAb HB-65. After staining, cells were washed and run on a BD LSR II flow cytometer, and virus infection frequencies, as measured by fractions of M2^+^ or NP^+^ cells, were quantified using FlowJo version 10.1 (Tree Star, Inc.).

### Intracellular viral RNA quantification.

Subconfluent 293T cells in six-well plates were infected with rPR8 at an MOI of 0.5 HA-expressing units (HAEU)/cell or were transfected with the following plasmids using jetPRIME (Polyplus): 3 μg of pHH21 vector plus 3 μg of pHH21::HA_vRNA_; RNP (500 ng each of pDZ::PB2, pDZ::PB1, pDZ::PA, and pDZ::NP) plus 1 μg of pHH21::HA_vRNA_. At 6 hpi and 24 h posttransfection, cells were harvested and RNA was extracted using an RNeasy minikit (Qiagen). cDNA of cellular mRNA and cDNA of HA vRNA were reverse transcribed with oligo(dT)_20_ or MBTuni (5′-ACGCGTGATCAGCAAAAGCAGG) reverse transcriptase (RT) primers using Superscript III (Invitrogen). Quantitative real-time PCR on cDNA was carried out using Power SYBR green PCR Master Mix (Thermo Fisher) on a QuantStudio 3 thermal cycler (Thermo Fisher). The strand-specific primers for quantitative real-time PCR of HA vRNA and GAPDH (glyceraldehyde-3-phosphate dehydrogenase) mRNA were as follows: for GAPDH317F, CTGGGGCTCACTTGAAAGG; for GAPDH388R, CAAACATGGGGGCATCAG); for HA, F (forward) (AAGGCAAACCTACTGGTCCTGTT) and R (reverse) (AATTGTTCGCATGGTAGCCTATAC). Delta threshold cycle (Δ*C_T_*) values for viral RNA were calculated by subtracting GAPDH *C_T_* values from HA *C_T_* values.

### Statistical analysis.

Unpaired, two-sided Student’s *t* tests were applied to the data shown in Fig. 1B, 2B, 3A, 3B, and 4F. An unpaired, two-sided Welch’s *t* test was applied to the data shown in Fig. 3D. All statistical analyses were performed with GraphPad Prism 7.0a.

## References

[1] Iuliano AD, Roguski KM, Chang HH, Muscatello DJ, Palekar R, Tempia S, Cohen C, Gran JM, Schanzer D, Cowling BJ, Wu P, Kyncl J, Ang LW, Park M, Redlberger-Fritz M, Yu H, Espenhain L, Krishnan A, Emukule G, van Asten L, Pereira da Silva S, Aungkulanon S, Buchholz U, Widdowson M-A, Bresee JS, Azziz-Baumgartner E, Cheng P-Y, Dawood F, Foppa I, Olsen S, Haber M, Jeffers C, MacIntyre CR, Newall AT, Wood JG, Kundi M, Popow-Kraupp T, Ahmed M, Rahman M, Marinho F, Sotomayor Proschle CV, Vergara Mallegas N, Luzhao F, Sa L, Barbosa-Ramírez J, Sanchez DM, Gomez LA, Vargas XB, et al 2017 Estimates of global seasonal influenza-associated respiratory mortality: a modelling study. Lancet 391:1285–1300. doi:10.1016/S0140-6736(17)33293-2. 29248255 PMC5935243

[2] Taubenberger JK, Morens DM 2010 Influenza: the once and future pandemic. Public Health Rep 125:16–26. PMC2862331 20568566

[3] Brooke CB 2014 Biological activities of “noninfectious” influenza A virus particles. Future Virol 9:41–51. doi:10.2217/fvl.13.118. 25067941 PMC4109409

[4] Brooke CB 2017 Population diversity and collective interactions during influenza virus infection. J Virol 91:e01164-17. doi:10.1128/JVI.01164-17. 28855247 PMC5660503

[5] McDonald SM, Nelson MI, Turner PE, Patton JT 2016 Reassortment in segmented RNA viruses: mechanisms and outcomes. Nat Rev Microbiol 14:448–460. doi:10.1038/nrmicro.2016.46. 27211789 PMC5119462

[6] Lowen AC 2017 Constraints, drivers, and implications of influenza A virus reassortment. Annu Rev Virol 4:105–121. doi:10.1146/annurev-virology-101416-041726. 28548881

[7] Taubenberger JK, Kash JC 2010 Influenza virus evolution, host adaptation, and pandemic formation. Cell Host Microbe 7:440–451. doi:10.1016/j.chom.2010.05.009. 20542248 PMC2892379

[8] Brooke CB, Ince WL, Wrammert J, Ahmed R, Wilson PC, Bennink JR, Yewdell JW 2013 Most influenza A virions fail to express at least one essential viral protein. J Virol 87:3155–3162. doi:10.1128/JVI.02284-12. 23283949 PMC3592173

[9] Brooke CB, Ince WL, Wei J, Bennink JR, Yewdell JW 2014 Influenza A virus nucleoprotein selectively decreases neuraminidase gene-segment packaging while enhancing viral fitness and transmissibility. Proc Natl Acad Sci U S A 111:16854–16859. doi:10.1073/pnas.1415396111. 25385602 PMC4250133

[10] Hirst GK 1973 Mechanism of influenza recombination. Virology 55:81–93. doi:10.1016/S0042-6822(73)81010-4. 4738051

[11] Hirst GK, Pons MW 1973 Mechanism of influenza recombination. Virology 56:620–631. doi:10.1016/0042-6822(73)90063-9. 4796550

[12] Russell AB, Trapnell C, Bloom JD 2018 Extreme heterogeneity of influenza virus infection in single cells. Elife 7:e32303. doi:10.7554/eLife.32303. 29451492 PMC5826275

[13] Dou D, Hernández-Neuta I, Wang H, Östbye H, Qian X, Thiele S, Resa-Infante P, Kouassi NM, Sender V, Hentrich K, Mellroth P, Henriques-Normark B, Gabriel G, Nilsson M, Daniels R 2017 Analysis of IAV replication and co-infection dynamics by a versatile RNA viral genome labeling method. Cell Rep 20:251–263. doi:10.1016/j.celrep.2017.06.021. 28683318

[14] White DO, Cheyne IM 1966 Early events in the eclipse phase of influenza and parainfluenza virus infection. Virology 29:49–59. doi:10.1016/0042-6822(66)90195-4. 4287026

[15] White DO, Day HM, Batchelder EJ, Cheyne IM, Wansbrough AJ 1965 Delay in the multiplication of influenza virus. Virology 25:289–302. doi:10.1016/0042-6822(65)90207-2. 14297216

[16] Ramig RF 1990 Superinfecting rotaviruses are not excluded from genetic interactions during asynchronous mixed infections in vitro. Virology 176:308–310. doi:10.1016/0042-6822(90)90260-X. 2158696

[17] Keirstead ND, Coombs KM 1998 Absence of superinfection exclusion during asynchronous reovirus infections of mouse, monkey, and human cell lines. Virus Res 54:225–235. doi:10.1016/S0168-1702(98)00023-9. 9696130 PMC7126977

[18] Nethe M, Berkhout B, van der Kuyl AC 2005 Retroviral superinfection resistance. Retrovirology 2:52. doi:10.1186/1742-4690-2-52. 16107223 PMC1224871

[19] Schaller T, Appel N, Koutsoudakis G, Kallis S, Lohmann V, Pietschmann T, Bartenschlager R 2007 Analysis of hepatitis C virus superinfection exclusion by using novel fluorochrome gene-tagged viral genomes. J Virol 81:4591–4603. doi:10.1128/JVI.02144-06. 17301154 PMC1900174

[20] Tscherne DM, Evans MJ, von Hahn T, Jones CT, Stamataki Z, McKeating JA, Lindenbach BD, Rice CM 2007 Superinfection exclusion in cells infected with hepatitis C virus. J Virol 81:3693–3703. doi:10.1128/JVI.01748-06. 17287280 PMC1866098

[21] Zou G, Zhang B, Lim P-Y, Yuan Z, Bernard KA, Shi P-Y 2009 Exclusion of West Nile virus superinfection through RNA replication. J Virol 83:11765–11776. doi:10.1128/JVI.01205-09. 19726510 PMC2772679

[22] Zhang X-F, Sun R, Guo Q, Zhang S, Meulia T, Halfmann R, Li D, Qu F 2017 A self-perpetuating repressive state of a viral replication protein blocks superinfection by the same virus. PLoS Pathog 13:e1006253. doi:10.1371/journal.ppat.1006253. 28267773 PMC5357057

[23] Laliberte JP, Moss B 2014 A novel mode of poxvirus superinfection exclusion that prevents fusion of the lipid bilayers of viral and cellular membranes. J Virol 88:9751–9768. doi:10.1128/JVI.00816-14. 24920806 PMC4136360

[24] Folimonova SY 2012 Superinfection exclusion is an active virus-controlled function that requires a specific viral protein. J Virol 86:5554–5561. doi:10.1128/JVI.00310-12. 22398285 PMC3347309

[25] Simon KO, Cardamone JJ, Whitaker-Dowling PA, Youngner JS, Widnell CC 1990 Cellular mechanisms in the superinfection exclusion of vesicular stomatitis virus. Virology 177:375–379. doi:10.1016/0042-6822(90)90494-C. 2162110

[26] Ludlow M 2005 Measles virus superinfection immunity and receptor redistribution in persistently infected NT2 cells. J Gen Virol 86:2291–2303. doi:10.1099/vir.0.81052-0. 16033977

[27] Huang I-C, Li W, Sui J, Marasco W, Choe H, Farzan M 2008 Influenza A virus neuraminidase limits viral superinfection. J Virol 82:4834–4843. doi:10.1128/JVI.00079-08. 18321971 PMC2346733

[28] Sobel Leonard A, McClain MT, Smith GJD, Wentworth DE, Halpin RA, Lin X, Ransier A, Stockwell TB, Das SR, Gilbert AS, Lambkin-Williams R, Ginsburg GS, Woods CW, Koelle K, Illingworth CJR 2017 The effective rate of influenza reassortment is limited during human infection. PLoS Pathog 13:e1006203. doi:10.1371/journal.ppat.1006203. 28170438 PMC5315410

[29] Xue KS, Stevens-Ayers T, Campbell AP, Englund JA, Pergam SA, Boeckh M, Bloom JD 2017 Parallel evolution of influenza across multiple spatiotemporal scales. Elife 6:e26875. doi:10.7554/eLife.26875. 28653624 PMC5487208

[30] Holmes EC, Ghedin E, Miller N, Taylor J, Bao Y, St George K, Grenfell BT, Salzberg SL, Fraser CM, Lipman DJ, Taubenberger JK 2005 Whole-genome analysis of human influenza A virus reveals multiple persistent lineages and reassortment among recent H3N2 viruses. PLoS Biol 3:e300. doi:10.1371/journal.pbio.0030300. 16026181 PMC1180517

[31] Rambaut A, Pybus OG, Nelson MI, Viboud C, Taubenberger JK, Holmes EC 2008 The genomic and epidemiological dynamics of human influenza A virus. Nature 453:615–619. doi:10.1038/nature06945. 18418375 PMC2441973

[32] Nelson MI, Edelman L, Spiro DJ, Boyne AR, Bera J, Halpin R, Ghedin E, Miller MA, Simonsen L, Viboud C, Holmes EC 2008 Molecular epidemiology of A/H3N2 and A/H1N1 influenza virus during a single epidemic season in the United States. PLoS Pathog 4:e1000133. doi:10.1371/annotation/1391941e-93d3-48d3-8c9a-b7c6d98f9527. 18725925 PMC2495036

[33] Nelson MI, Viboud C, Simonsen L, Bennett RT, Griesemer SB, St George K, Taylor J, Spiro DJ, Sengamalay NA, Ghedin E, Taubenberger JK, Holmes EC 2008 Multiple reassortment events in the evolutionary history of H1N1 influenza A virus since 1918. PLoS Pathog 4:e1000012. doi:10.1371/annotation/1391941e-93d3-48d3-8c9a-b7c6d98f9527. 18463694 PMC2262849

[34] Westgeest KB, Russell CA, Lin X, Spronken MIJ, Bestebroer TM, Bahl J, van Beek R, Skepner E, Halpin RA, de Jong JC, Rimmelzwaan GF, Osterhaus ADME, Smith DJ, Wentworth DE, Fouchier RAM, de Graaf M, Garcia-Sastre A 2014 Genomewide analysis of reassortment and evolution of human influenza A(H3N2) viruses circulating between 1968 and 2011. J Virol 88:2844–2857. doi:10.1128/JVI.02163-13. 24371052 PMC3958060

[35] Maljkovic Berry I, Melendrez MC, Li T, Hawksworth AW, Brice GT, Blair PJ, Halsey ES, Williams M, Fernandez S, Yoon I-K, Edwards LD, Kuschner R, Lin X, Thomas SJ, Jarman RG 2016 Frequency of influenza H3N2 intra-subtype reassortment: attributes and implications of reassortant spread. BMC Biol 14:117. doi:10.1186/s12915-016-0337-3. 28034300 PMC5200972

[36] Marshall N, Priyamvada L, Ende Z, Steel J, Lowen AC 2013 Influenza virus reassortment occurs with high frequency in the absence of segment mismatch. PLoS Pathog 9:e1003421. doi:10.1371/journal.ppat.1003421. 23785286 PMC3681746

[37] Fukuyama S, Katsura H, Zhao D, Ozawa M, Ando T, Shoemaker JE, Ishikawa I, Yamada S, Neumann G, Watanabe S, Kitano H, Kawaoka Y 2015 Multi-spectral fluorescent reporter influenza viruses (Color-flu) as powerful tools for in vivo studies. Nat Commun 6:6600. doi:10.1038/ncomms7600. 25807527 PMC4389232

[38] Martin K, Helenius A 1991 Nuclear transport of influenza virus ribonucleoproteins: the viral matrix protein (M1) promotes export and inhibits import. Cell 67:117–130. doi:10.1016/0092-8674(91)90576-K. 1913813

[39] Ohkuma S, Poole B 1978 Fluorescence probe measurement of the intralysosomal pH in living cells and the perturbation of pH by various agents. Proc Natl Acad Sci U S A 75:3327–3331. doi:10.1073/pnas.75.7.3327. 28524 PMC392768

[40] Desmyter J, Melnick JL, Rawls WE 1968 Defectiveness of interferon production and of rubella virus interference in a line of African green monkey kidney cells (Vero). J Virol 2:955–961. 4302013 10.1128/jvi.2.10.955-961.1968 PMC375423

[41] Emeny JM, Morgan MJ 1979 Regulation of the interferon system: evidence that Vero cells have a genetic defect in interferon production. J Gen Virol 43:247–252. doi:10.1099/0022-1317-43-1-247. 113494

[42] Hensley SE, Das SR, Gibbs JS, Bailey AL, Schmidt LM, Bennink JR, Yewdell JW 2011 Influenza A virus hemagglutinin antibody escape promotes neuraminidase antigenic variation and drug resistance. PLoS One 6:e15190. doi:10.1371/journal.pone.0015190. 21364978 PMC3043005

[43] Fonville JM, Marshall N, Tao H, Steel J, Lowen AC 2015 Influenza virus reassortment is enhanced by semi-infectious particles but can be suppressed by defective interfering particles. PLoS Pathog 11:e1005204. doi:10.1371/journal.ppat.1005204. 26440404 PMC4595279

[44] Dixit E, Boulant S, Zhang Y, Lee ASY, Odendall C, Shum B, Hacohen N, Chen ZJ, Whelan SP, Fransen M, Nibert ML, Superti-Furga G, Kagan JC 2010 Peroxisomes are signaling platforms for antiviral innate immunity. Cell 141:668–681. doi:10.1016/j.cell.2010.04.018. 20451243 PMC3670185

[45] Wack A, Terczyńska-Dyla E, Hartmann R 2015 Guarding the frontiers: the biology of type III interferons. Nat Immunol 16:802–809. doi:10.1038/ni.3212. 26194286 PMC7096991

[46] Odendall C, Kagan JC 2015 The unique regulation and functions of type III interferons in antiviral immunity. Curr Opin Virol 12:47–52. doi:10.1016/j.coviro.2015.02.003. 25771505 PMC4470718

[47] Diefenbacher M, Sun J, Brooke CB 2018 The parts are greater than the whole: the role of semi-infectious particles in influenza A virus biology. Curr Opin Virol 33:42–46. doi:10.1016/j.coviro.2018.07.002. 30053722 PMC6642613

[48] Sanjuán R 2017 Collective infectious units in viruses. Trends Microbiol 25:402–412. doi:10.1016/j.tim.2017.02.003. 28262512 PMC5837019

