# Supplementary figures and images for: Influenza A Virus Superinfection Potential Is Regulated by Viral Genomic Heterogeneity
Source: mBio. 2018 Oct 30;9(5):e01761-18. doi: 10.1128/mBio.01761-18 (PMC6212824; doi:10.1128/mBio.01761-18)

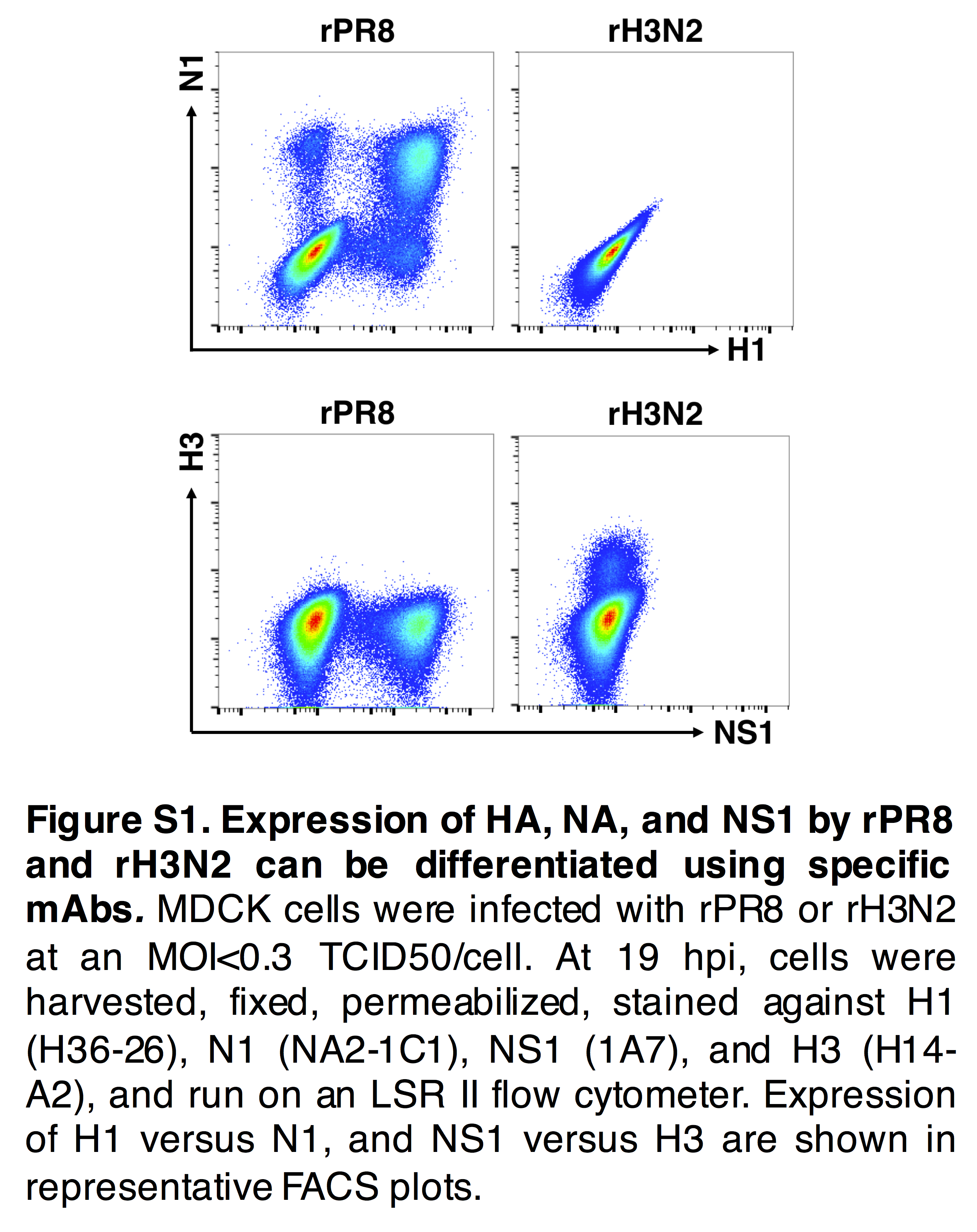

Supplement: FIG S1 [file mbo005184144sf1.tif]

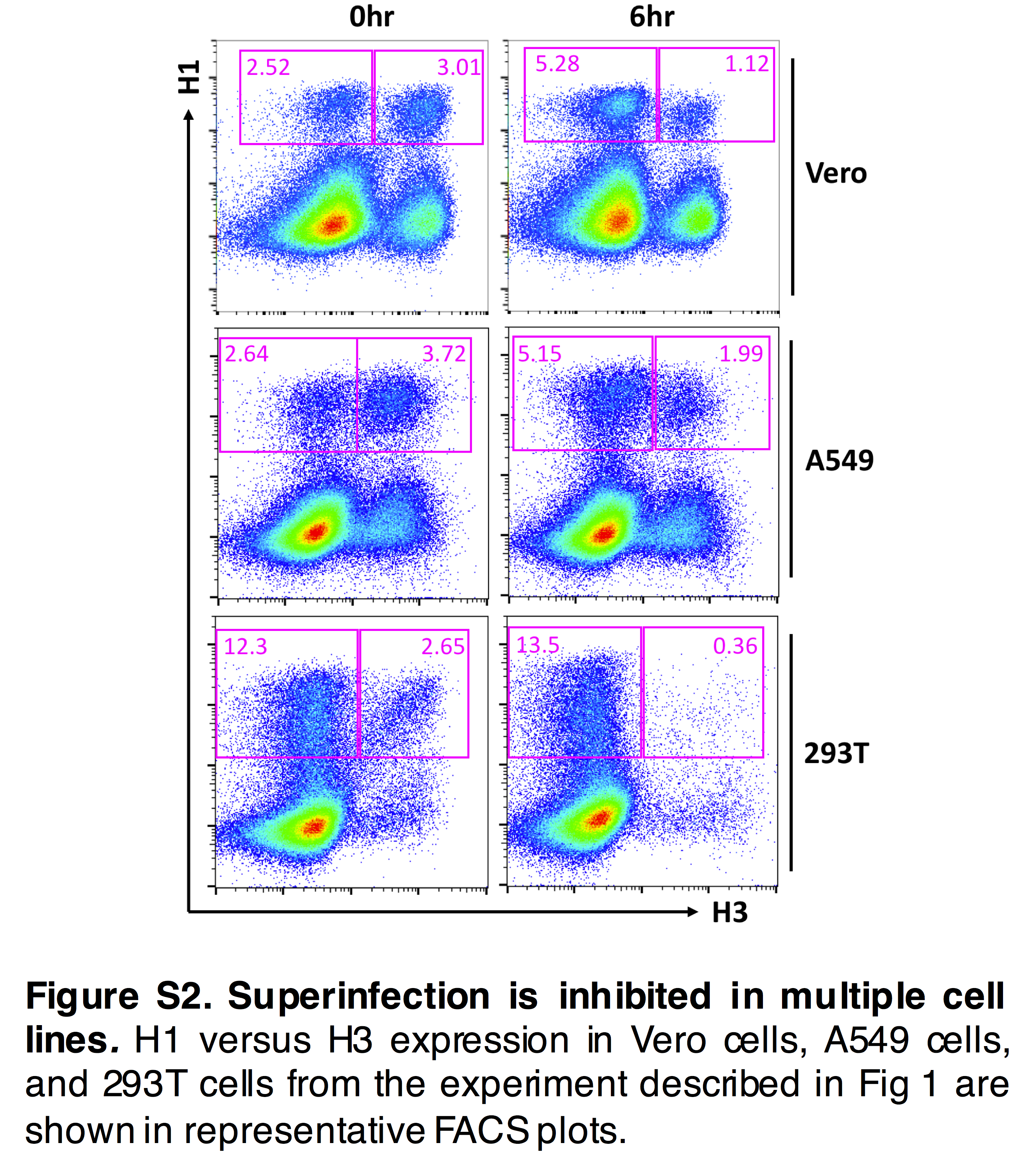

Supplement: FIG S2 [file mbo005184144sf2.tif]

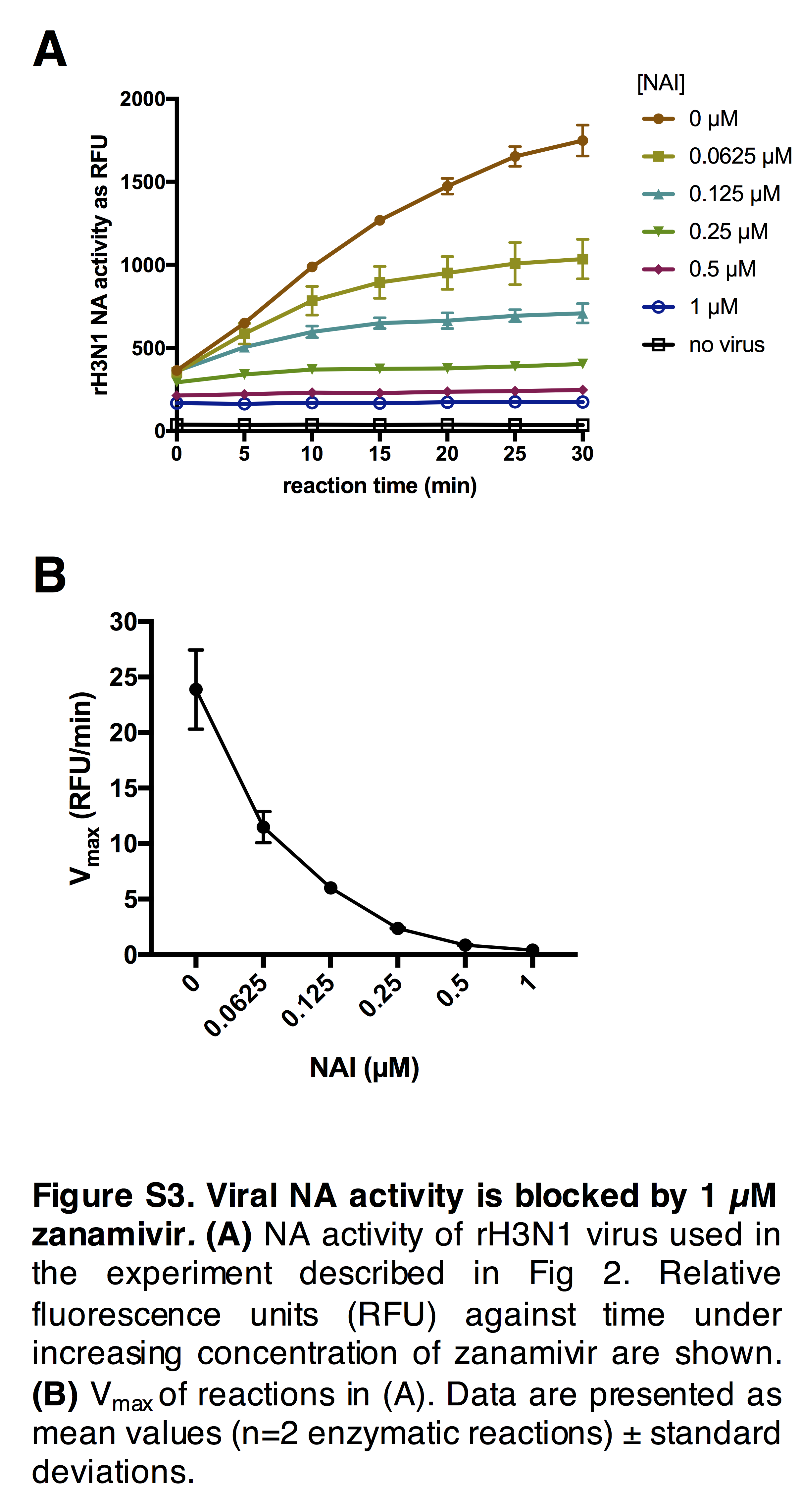

Supplement: FIG S3 [file mbo005184144sf3.tif]

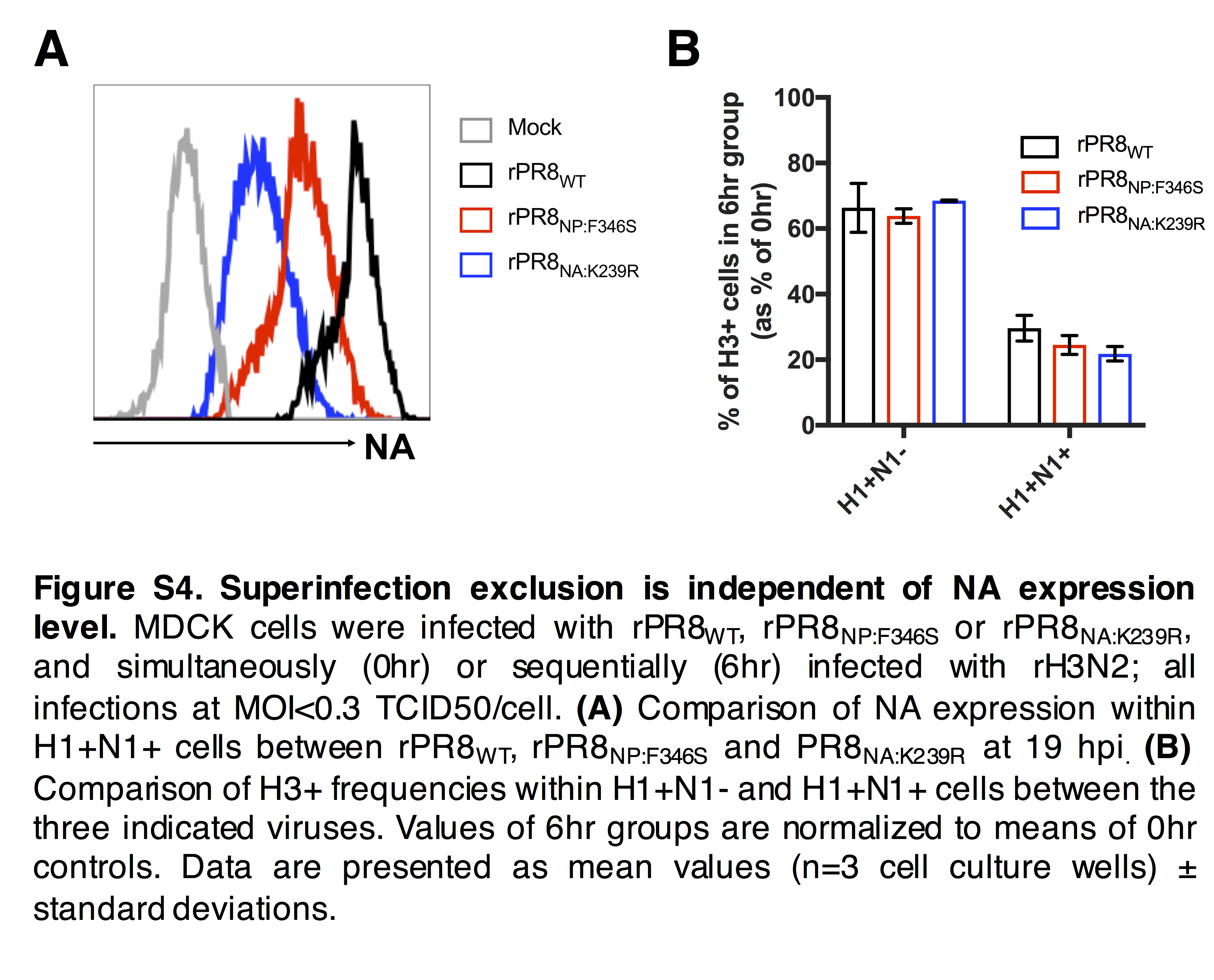

Supplement: FIG S4 [file mbo005184144sf4.tif]

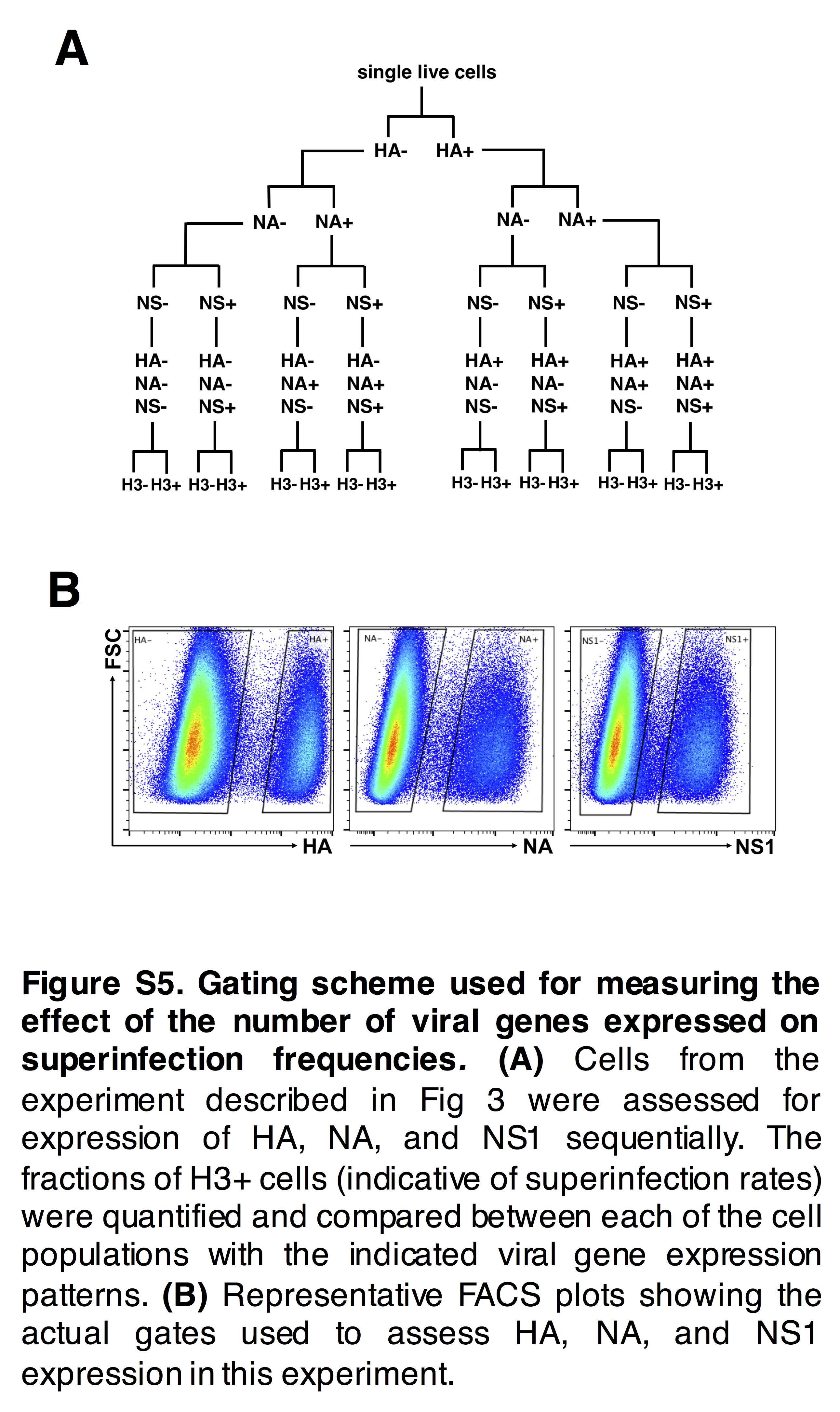

Supplement: FIG S5 [file mbo005184144sf5.tif]

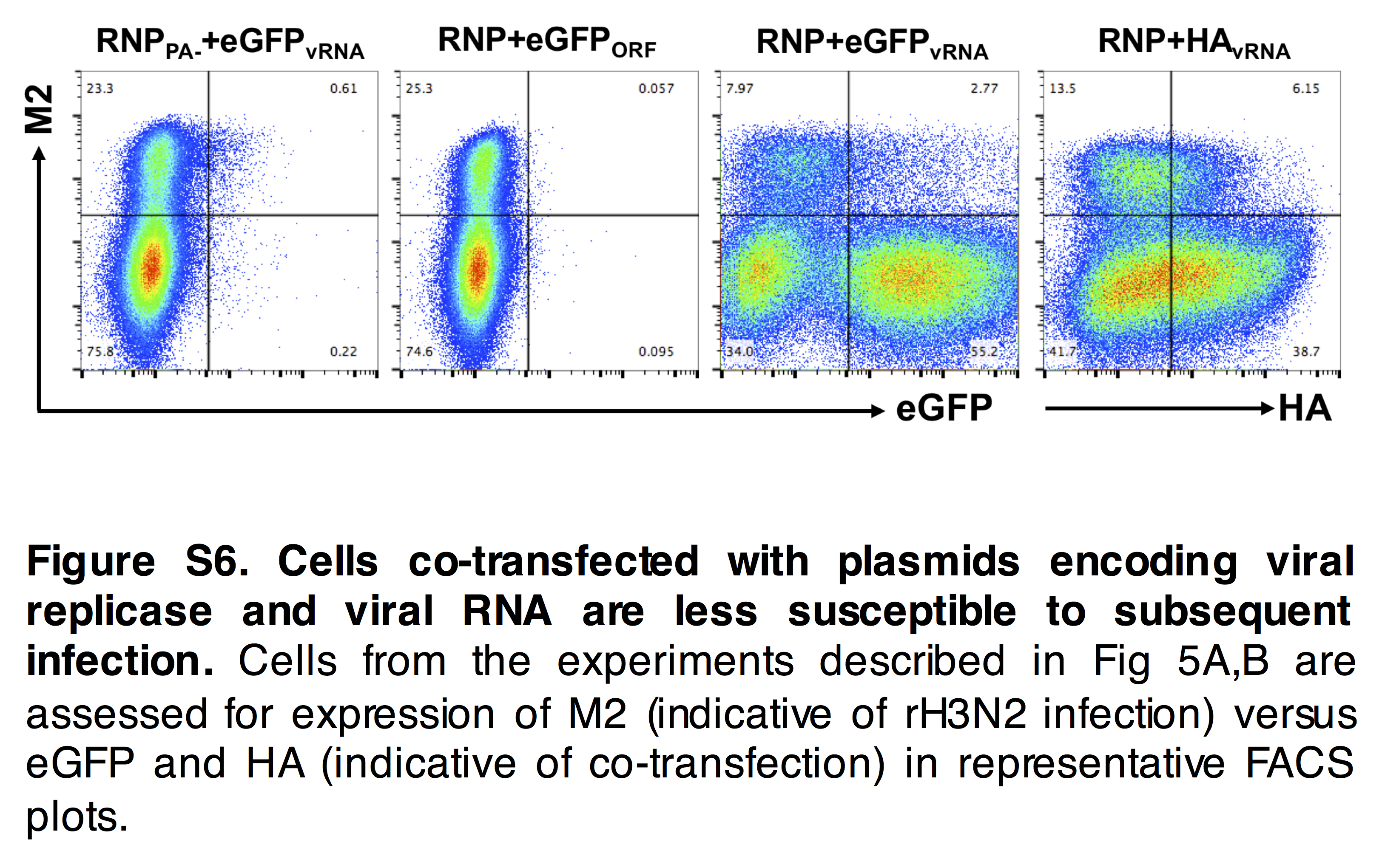

Supplement: FIG S6 [file mbo005184144sf6.tif]

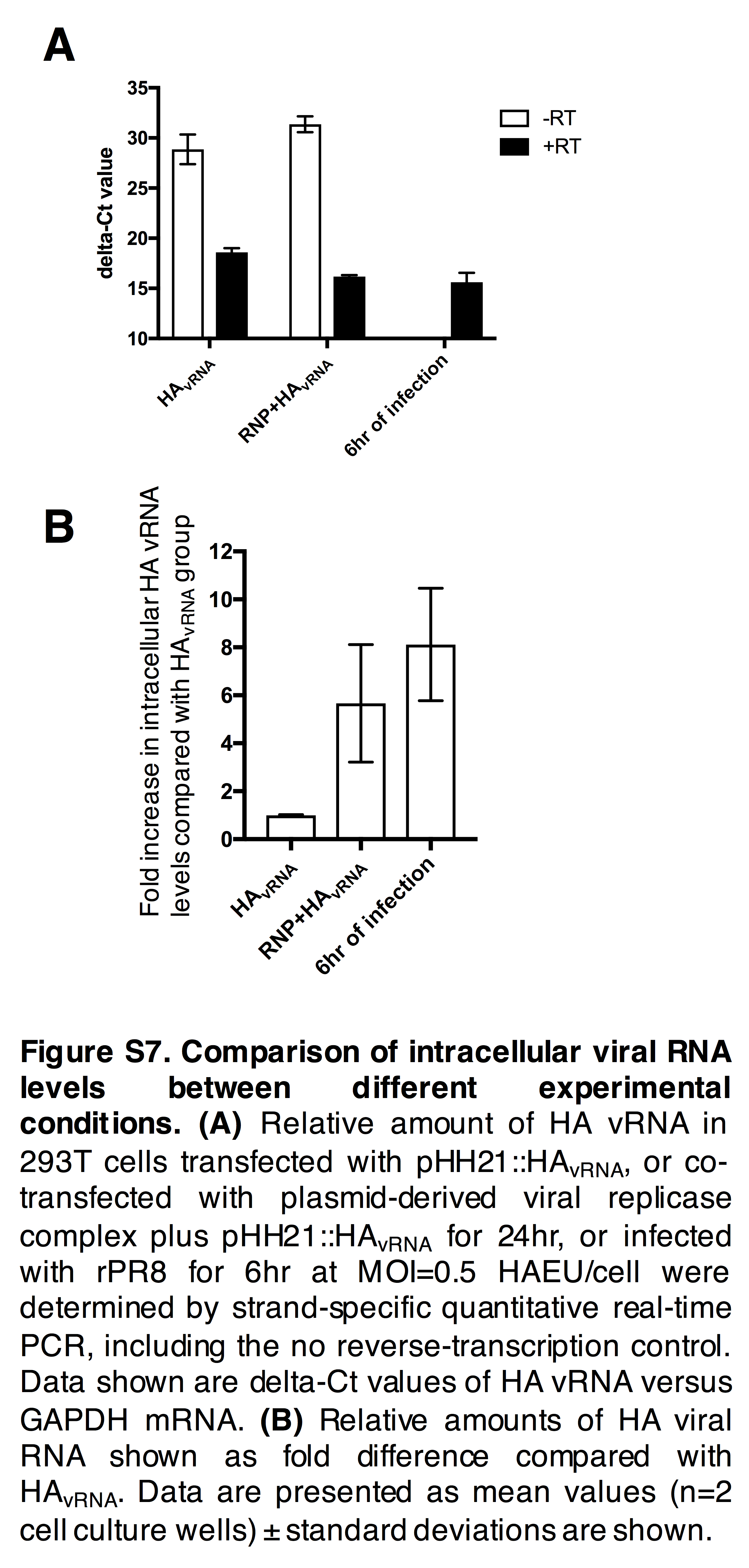

Supplement: FIG S7 [file mbo005184144sf7.tif]

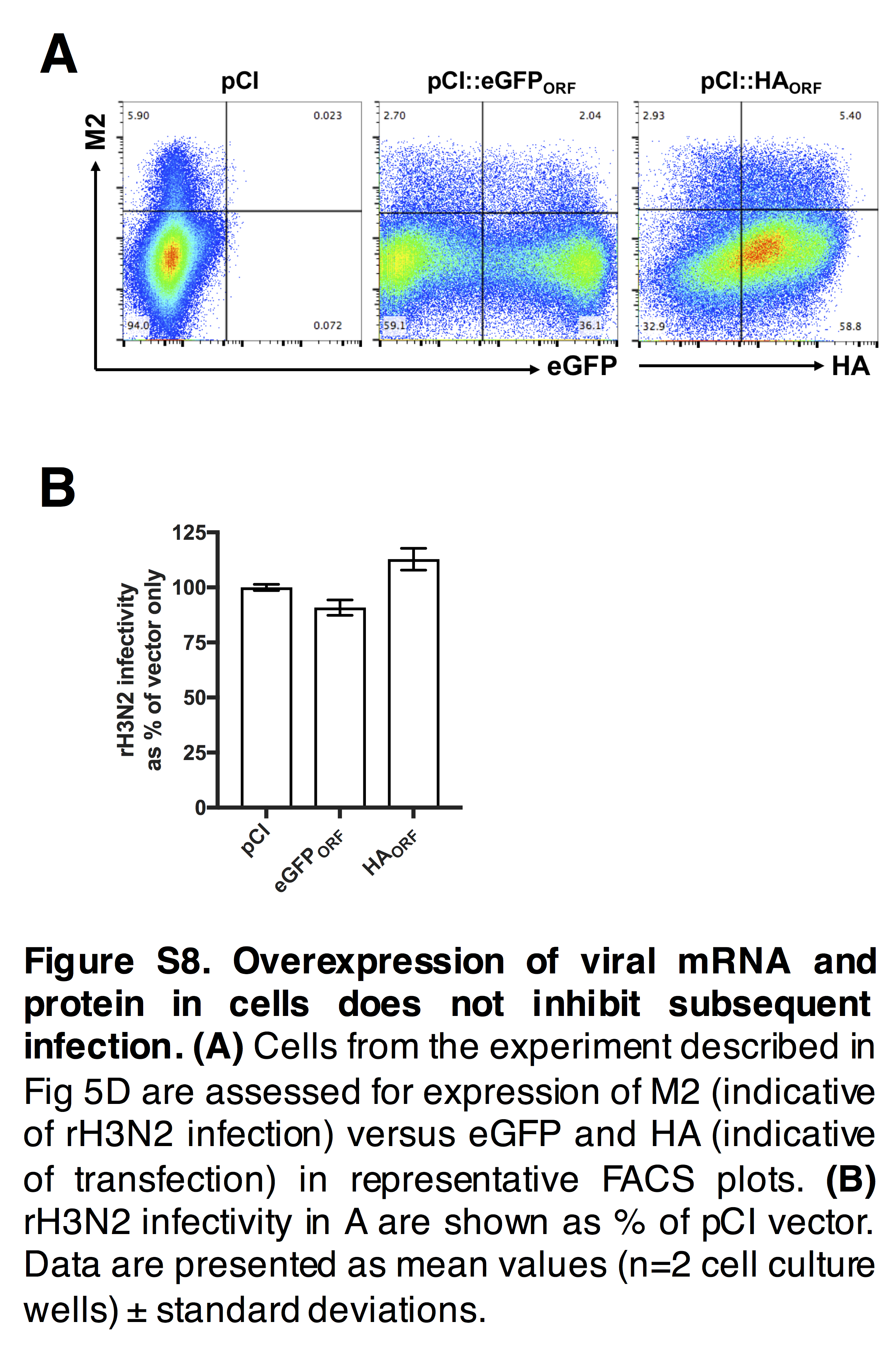

Supplement: FIG S8 [file mbo005184144sf8.tif]
